# Phenotypic Characteristics Associated with Virulence of Clinical Isolates from the *Sporothrix* Complex

**DOI:** 10.1155/2015/212308

**Published:** 2015-04-19

**Authors:** Rodrigo Almeida-Paes, Luã Cardoso de Oliveira, Manoel Marques Evangelista Oliveira, Maria Clara Gutierrez-Galhardo, Joshua Daniel Nosanchuk, Rosely Maria Zancopé-Oliveira

**Affiliations:** ^1^Laboratório de Micologia, Instituto Nacional de Infectologia Evandro Chagas, Fundação Oswaldo Cruz, Avenida Brasil 4365, 21045-900 Manguinhos, RJ, Brazil; ^2^Laboratório de Dermatologia Infecciosa, Instituto Nacional de Infectologia Evandro Chagas, Fundação Oswaldo Cruz, Avenida Brasil 4365, 21045-900 Manguinhos, RJ, Brazil; ^3^Department of Medicine (Division of Infectious Diseases), Albert Einstein College of Medicine, 1300 Morris Park Avenue, Bronx, NY 10461, USA; ^4^Department of Microbiology and Immunology, Albert Einstein College of Medicine, 1300 Morris Park Avenue, Bronx, NY 10461, USA

## Abstract

The *Sporothrix* complex members cause sporotrichosis, a subcutaneous mycosis with a wide spectrum of clinical manifestations. Several specific phenotypic characteristics are associated with virulence in many fungi, but studies in this field involving the *Sporothrix* complex species are scarce. Melanization, thermotolerance, and production of proteases, catalase, and urease were investigated in 61 *S. brasiliensis*, one *S. globosa*, and 10 *S. schenckii* strains. The *S. brasiliensis* strains showed a higher expression of melanin and urease compared with *S. schenckii*. These two species, however, presented similar thermotolerances. Our *S. globosa* strain had low expression of all studied virulence factors. The relationship between these phenotypes and clinical aspects of sporotrichosis was also evaluated. Strains isolated from patients with spontaneous regression of infection were heavily melanized and produced high urease levels. Melanin was also related to dissemination of internal organs and protease production was associated with HIV-coinfection. A murine sporotrichosis model showed that a *S. brasiliensis* strain with high expression of virulence factors was able to disseminate and yield a high fungal burden in comparison with a control *S. schenckii* strain. Our results show that virulence-related phenotypes are variably expressed within the *Sporothrix* complex species and might be involved in clinical aspects of sporotrichosis.

## 1. Introduction

Sporotrichosis is a subcutaneous cosmopolitan mycosis, with a high prevalence in Latin America, especially in Brazil, Peru, and Colombia [1]. The disease is also endemic in India, China, Japan, and South Africa [2]. In addition, in some countries, such as Italy [3], Portugal [4], and Greece [5], sporotrichosis sporadic clinical cases have been reported in recent years. This mycosis is caused by the members of the* Sporothrix* complex [6], especially by the species* Sporothrix brasiliensis*,* S. globosa*,* S. mexicana*,* S. luriei*, and, classically,* S. schenckii* sensu stricto [7]. The major species involved in cases of human or animal sporotrichosis are* S. brasiliensis*,* S. schenckii*, and* S. globosa* [2]. A few cases, however, are caused by* S. mexicana* [8] and* S. pallida *[9] and by the rare pathogen* S. luriei* [10].

A wide spectrum of clinical manifestations is observed in sporotrichosis, ranging from cutaneous, subcutaneous, and disseminated cutaneous forms to pulmonary or systemic presentations [2]. The virulence factors related to the* Sporothrix* complex species, which could be related to these manifestations, are not well known, mainly because the historical lack of genetic information. Also their teleomorphs remain unknown, which hinders the application of classic genetic approaches in this complex [11]. However, it is believed that the production of certain proteins or glycoproteins, melanin, and ergosterol peroxide as well as the thermotolerance of the fungi could influence clinical manifestations of sporotrichosis [2], as observed for diseases caused by other pathogenic fungi, such as* Cryptococcus neoformans* [12],* Histoplasma capsulatum* [13],* Coccidioides posadasii* [14],* Aspergillus fumigatus* [15], and* Paracoccidioides brasiliensis* [16], among several other pathogenic fungi [17].

Melanin is an important virulence factor for many pathogenic fungi. The presence of this pigment in the cell wall increases fungal survival under harsh environmental and parasitic conditions [18, 19]. Both morphologic forms of* Sporothrix* spp. are able to produce at least one of three types of melanin (DHN-melanin, eumelanin, or pyomelanin) and it has been observed that* Sporothrix* melanins protect the fungus against Amphotericin B and nitrogen-derived oxidants [20]. Other putative virulence factors of* Sporothrix* spp., such as thermotolerance, are fundamental for the survival of* Sporothrix* spp. in parasitism [2]. For instance, strains that grow at 35°C but not at 37°C are unlikely to disseminate through the lymphatic system of the host [21].

Other presumed factors not yet rigorously investigated may also be important for the pathogenesis of sporotrichosis. For example, urease and proteases are important in other fungi such as* C. neoformans* [22] and* C. posadasii* [23]. Protease production by* C. posadasii* facilitates evasion of host cell phagocytosis [24]. In* C. neoformans*, proteases appear to be important during the invasion through the brain-blood barrier [25]. Therefore, the study of* Sporothrix *putative virulence factors is necessary for a better understanding of the biology, physiology, and pathogenicity of these cryptic species [11]. Hence, the aim of this study was to evaluate the production of putative virulence factors, such as melanin, proteases, catalase, and urease as well as thermotolerance of* Sporothrix* spp., and correlate them with the different clinical forms and the species of this cryptic complex.

## 2. Materials and Methods

### 2.1. Strains

In this study, we included 71* Sporothrix* spp. strains obtained from patients with sporotrichosis in three different Brazilian states: Rio de Janeiro (*n* = 64), Espírito Santo (*n* = 5), and Rio Grande do Sul (*n* = 2). The use of these strains and deidentified patient data was approved by the Institutional Ethics Review Board of the Fundação Oswaldo Cruz. Additionally, a control* S. schenckii* strain (ATCC 16345) isolated in Baltimore, USA, was also included. Sequencing of the partial calmodulin gene [7] was performed to classify the strains as* S. schenckii* (*n* = 10),* S. globosa* (*n* = 1), or* S. brasiliensis* (*n* = 61). Information about clinical origin of samples, clinical form of sporotrichosis, year of isolation, patient's treatment for sporotrichosis, and HIV status of the patients was obtained for all but the control (ATCC 16345) strain.

### 2.2. Melanin Production

Melanization was assessed by growth on Sabouraud Dextrose Agar (SDA) and in a minimal medium (MM) that consists of 15 mM glucose, 10 mM MgSO_4_, 29.4 mM K_2_HPO_4_, 13 mM glycine, and 3.0 mM thiamine, pH 5.5. Plates were incubated at 25°C and observed daily for melanin production. The degree of melanization at 30 days of incubation was scored as (−) if the colonies were white, (+) if the colonies were pale brown, (++) if the colonies were dark brown, and (+++) if black colonies were black.

### 2.3. Thermotolerance

The ability of* Sporothrix* strains to grow at 30°C and 37°C was quantified as described previously [26]. In brief, the strains were plated on SDA and incubated at 30°C and 37°C during 15 and 21 days. At these times, the diameter of the colonies on both temperatures was measured and the percent growth inhibition (%GI) was calculated as follows:

(1) $$\begin{array}{c} \%\text{GI}=\frac{D_{30}-D_{37}}{D_{30}}\times 100, \end{array}$$

where *D*_30_ and *D*_37_ are the diameters of the colonies at 30 and 37°C, respectively. To further verify if* Sporothrix* melanization influences its thermotolerance, two approaches were made. First, the degree of melanization was correlated to the % GI of each strain. Additionally, four* S. brasiliensis*, one* S. globosa*, and two* S. schenckii* strains were plated on SDA supplemented with tricyclazole 8 mg/L, L-DOPA 1 mM, or L-tyrosine 10 mM, which are, respectively, an inhibitor of DHN-melanin synthesis, a precursor for eumelanin synthesis, and a precursor for pyomelanin synthesis.

### 2.4. Azoalbumin Degradation Test

The proteolytic activity of the isolates was studied as previously described for* C. neoformans* [27]. Briefly, yeast cells of all isolates were inoculated in Petri dishes containing MM supplemented with 0.1% azoalbumin. These plates were incubated at 37°C for fifteen days. After incubation, each plate was inspected for the production of an azoalbumin degradation halo around the* Sporothrix* colonies. The diameter of the colony and the diameter of the azoalbumin degradation halo were measured and the *P*/*z* value was calculated as the ratio between the two diameters, respectively. Isolates that presented proteolytic activity at 37°C were further inoculated in MM supplemented with 0.1% azoalbumin with subsequent incubation at 30°C for comparison between the proteolytic activity in yeast and filamentous forms, respectively.

### 2.5. Gelatin Liquefaction Assay

To further investigate extracellular proteases, the strains were also inoculated in nutrient gelatin (BBL, Becton, Dickinson and Company, Sparks, USA), a medium for the determination of gelatin degradation by nonfastidious microorganisms. An inoculum of 1 × 10^6^ yeast cells was inoculated directly down the center of the medium. After 14 days of incubation at 35°C, tubes were transferred to a refrigerator for 18 hours. After this time period, tubes were inverted to test for solidification or liquefaction. A control tube without fungal inoculation was used as solidification control and* C. neoformans* ATCC 24067 was used as a liquefaction control [27].

### 2.6. Semiquantitative Catalase Test

The method described to divide mycobacteria into low or high catalase producers [28] was applied to the* Sporothrix* strains, with minor modifications. In brief, screw-cap tubes containing Brain Heart Infusion Agar were inoculated with 200 *μ*L of a suspension of yeast* Sporothrix* cells corresponding to the 0.5 McFarland standard. The media were incubated for 7 days at 35°C. After this incubation, 1 mL of a freshly prepared 1 : 1 mixture of 10% Tween 80 and 30% hydrogen peroxide was added to the cultures. The time to bubble production was observed, as well as the size in millimeters of the column of bubbles after 5 minutes at room temperature. Uninoculated medium was used as a negative control. Strains were classified as low or high catalase producers if the size of the column of bubbles was less or higher than 45 mm, respectively.

### 2.7. Urease Production

To verify the production of urease, the different isolates were cultured on Christensen urea broth [29]. Suspensions of yeast cells of each isolate equivalent to the 2.0 McFarland scale were used for inoculation. A volume of 500 *μ*L of the suspension was inoculated in 4.5 mL of Christensen urea broth and the tubes were then incubated at 37°C. At the end of four and seven days of incubation, the tubes were centrifuged and a volume of 100 *μ*L of the supernatant transferred to a 96 well polystyrene flat bottom plate (Corning, Tewksbury, USA) in triplicate.* Trichophyton rubrum* ATCC 28189 and* Trichophyton mentagrophytes* ATCC 62905 were used as negative and positive controls, respectively [30]. The absorbance of samples was read on a spectrophotometer Biotek model Epoch at 559 nm, which corresponds to the absorption peaks of positive samples obtained after scanning the spectrum of the supernatant of the above controls from 400 to 700 nm. To verify the influence of fungal growth on the absorbance, the CFU/mL was determined for the* Sporothrix* strains as previously described [20] before harvesting the supernatants for absorbance measurements.

### 2.8. Virulence Assay

To explore if the differences observed on the phenotypic assays were linked to fungal virulence, the* S. brasiliensis* IPEC 26449 producing high levels of the studied virulence factors and* S. schenckii* ATCC 16345 were inoculated intraperitoneally into six- to eight-week-old C57/Bl6 male mice. This work was approved by the Animal Use Committee of the Albert Einstein College of Medicine. Ten mice were used per strain, an inoculum of 5 × 10^7^ yeast was used in five animals, and the other five were inoculated with 1 × 10^8^ yeasts. As a control, five mice were inoculated with PBS. The animals were housed in cages kept in a room with controlled temperature, light, and humidity. Food and water were provided* ad libitum*. After 20 days, the animals were euthanatized by CO_2_ inhalation using a chamber coupled to a compressed CO_2_ cylinder (USP grade A). After this procedure, the lungs, liver, and spleen were removed. The organs were weighted, macerated and serially diluted in PBS. The dilutions were plated on SDA with 400 mg/L chloramphenicol for determining the number of CFU/g per organ after incubation at 25°C during 7 days.

### 2.9. Statistical Analysis

Analyses were performed using GraphPad Prism 5.0 software. Nonparametric tests were used to compare groups. A *P* < 0.05 was considered significant.

## 3. Results

### 3.1. Melanization

**Figure 1:**
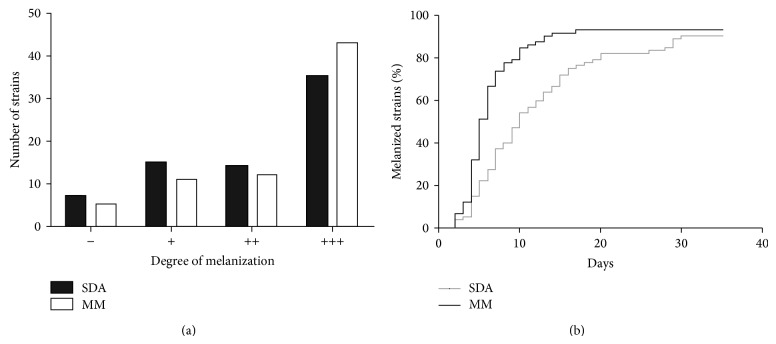
Production of melanin by members of the* Sporothrix* complex is dependent on the available nutrients in the microenvironment. (a) Degree of melanization and (b) melanization times of 72* Sporothrix* strains in Sabouraud Dextrose Agar (SDA) and minimal medium (MM).

**Figure 2:**
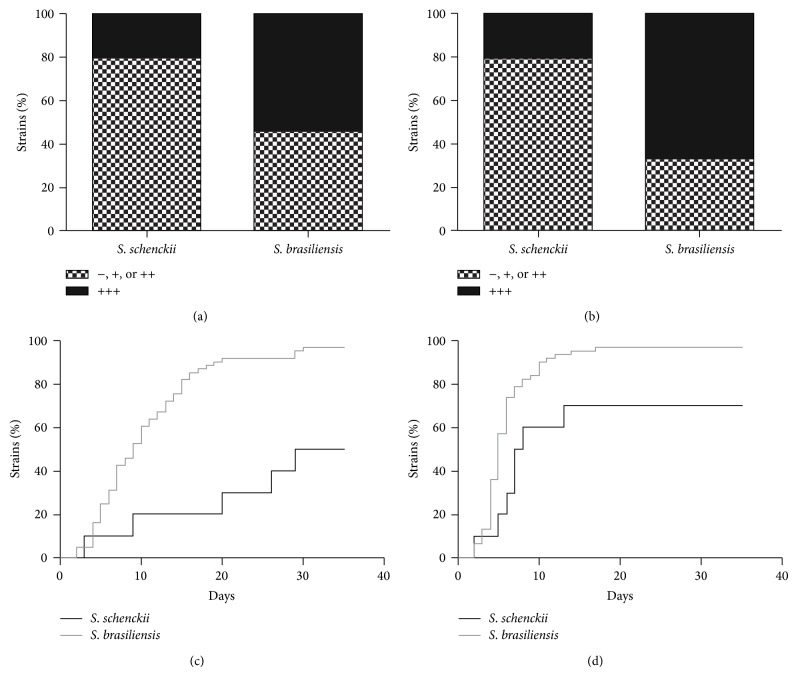
Differential production of melanin by* S. brasiliensis* and* S. schenckii* sensu stricto. ((a), (b)) Percent melanized strains and ((c), (d)) melanization times of the two species in ((a), (c)) and Sabouraud Dextrose Agar and in ((b), (d)) minimal medium.

The strains presented different degrees of melanization, which also varied according to the media used. In general, cultures produced more DHN-melanin in MM than in SDA at 30 days of incubation (Figure 1(a)), although this difference was not significant (*P* = 0.59). However, melanin production in MM is significantly faster than in SDA (*P* = 0.0002), with median times to start the melanization process in SDA and MM of 10 and 5 days, respectively (Figure 1(b)). In addition, some strains, such as* S. brasiliensis* IPEC 25758, become pigmented after only a few days but do not produce heavily melanized cultures, even at 30 days of growth. By contrast, other strains, such as* S. brasiliensis* IPEC 29039 or* S. schenckii* IPEC 27722, take a longer time to melanize, but when they do, they produce very dark colonies. One* S. schenckii* and 30* S. brasiliensis* strains produced heavily melanized colonies on both tested media. On the other hand, three* S. schenckii* and one* S. brasiliensis* strain were unable to produce visible dark pigment on both media by 30 days of growth at 25°C. In general, more* S. brasiliensis* strains produced heavily melanized colonies, compared to* S. schenckii* (Figures 2(a) and 2(b)), on both SDA (54% and 20% resp.) and MM (67% and 20%, resp.). According to Fisher's exact test, this difference was significant on the MM (*P* = 0.011), but not on SDA (*P* = 0.085). Also, the time for* S. brasiliensis* cultures visibly appearing pigmented was significantly lower than for* S. schenckii* cultures, on both SDA (Figure 2(c)) and MM (Figure 2(d)), with *P* values of 0.0002 and 0.011, respectively. The* S. globosa* IPEC 27135 strain produced lightly melanized colonies on SDA and MM, at 28 and 13 days, respectively. The control* S. schenckii* ATCC 16345 strain did not produce melanin under the studied conditions.

### 3.2. Thermotolerance

**Figure 3:**
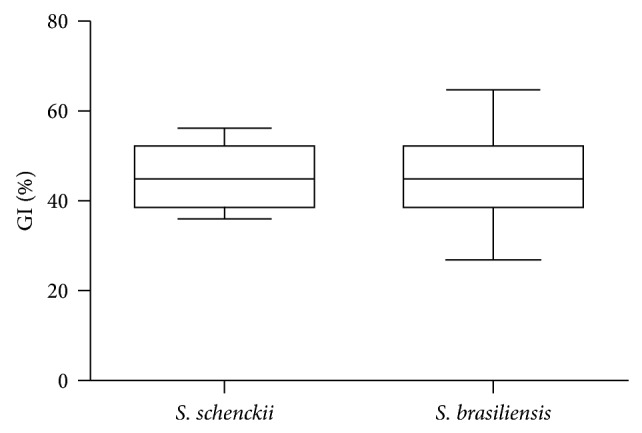
Box-plot diagrams showing similar thermotolerances between* S. brasiliensis* and* S. schenckii* sensu stricto observed at 21 days of growth in Sabouraud Dextrose Agar.

**Figure 4:**
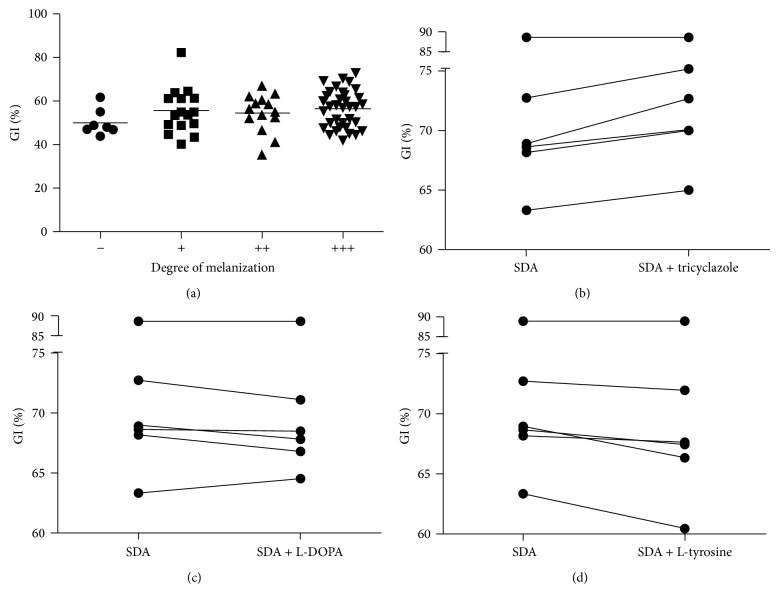
Correlation between melanization and thermotolerance in the* Sporothrix* complex. (a) The growth inhibition of the strains is not dependent on their levels of DHN-melanin. (b) Tricyclazole, a DHN-melanin inhibitor, is able to enhance fungal growth inhibition. (c) L-DOPA and (d) L-tyrosine, precursors for eumelanin and pyomelanin, respectively, are able to reduce growth inhibition in some strains.

As expected, the* S. globosa* IPEC 27135 strain was highly inhibited when cultured at 37°C, with a %GI of 86.7% and 82.4% at 15 and 21 days, respectively. The %GI of* S. brasiliensis* strains ranged from 32.3 to 71% at 15 days and from 35.1 to 72.9% at 21 days, with mean values of 52.5 ± 10.2% and 54.8 ± 8.5%, respectively. For* S. schenckii* isolates, ranges were 41.4–65.9% and 43.9–64.5% and the mean values were 52.7 ± 8.7% and 55 ± 6.9% at 15 and 21 days of growth at 37°C, respectively. There was no difference between* S. brasiliensis* and* S. schenckii* %GI at 15 or 21 days of incubation (Figure 3), with a *P* value of 0.90 for both times. Additionally, as presented in Figure 4(a), no correlation was seen between the %GI and the degree of melanization of the* Sporothrix* strains (*P* = 0.51 at 15 days and *P* = 0.35 at 21 days). When we tested the thermotolerance of representative strains in presence of tricyclazole, the %GI values increased in five strains and decreased in only one (Figure 4(b)). However, when the strains were cultured in presence of L-DOPA or L-tyrosine, conditions where the fungus can produce eumelanin or pyomelanin in addition to DHN-melanin, we observed that five strains have decreased %GI values, though the decrease was slight in two strains with L-DOPA and one with L-tyrosine. The decrease of %GI values in presence of L-DOPA (Figure 4(c)) was lower than in presence of L-tyrosine (Figure 4(d)).

### 3.3. Hydrolytic Enzymes

**Figure 5:**
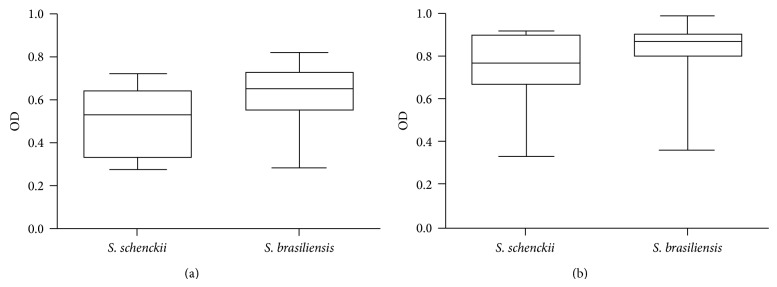
Urease production, as measured by the optical density (OD) at 559 nm, of the yeast-form of 72* Sporothrix* strains at (a) four and (b) seven days of incubation at 37°C in Christensen's urea broth.

No differences were observed between the productions of catalase by the 72 studied strains. All isolates produced bubbles almost immediately and were classified as high catalase producers. The azoalbumin test was able to detect proteolytic activity on the yeast phase of 15 (20.8%) strains, all from Rio de Janeiro state. The number of positive* S. brasiliensis* strains (*n* = 13; 21.3%) was not significantly different (*P* = 0.75) from* S. schenckii* (*n* = 2; 20%). Of these 15 positive strains, only three (all* S. brasiliensis*) were also able to produce an azoalbumin degradation halo in their mycelial phase at 30°C.  Table 1 shows the proteolytic activity, expressed as the *P*/*z* value, for the positive isolates at both tested temperatures. The gelatin liquefaction assay showed negative results, at both 30°C and 37°C, for all tested strains, including those that were able to degrade azoalbumin in the yeast phase. No proteolytic activity was observed for the control* S. schenckii* ATCC 16345 strain. Urease production was highly variable between isolates. Overall, most strains were able to degrade the urea presented on the culture medium. After seven days of incubation at 37°C, three strains (one* S. brasiliensis* and two* S. schenckii*) had no detectable urease activity. The differences in urease activity were not related to the fungal growth on the Christensen's urea broth, since all strains yielded 3.6 × 10^6^ to 5 × 10^6^ CFU/mL (*P* = 0.88). When the optical densities at 559 nm of culture supernatants were determined, we observed that* S. brasiliensis* produced more urease than* S. schenckii* (*P* = 0.007) at four days of incubation at 37°C (Figure 5(a)). Though it was lower, this difference was also significant (*P* = 0.027) at seven days of incubation (Figure 5(b)).

### 3.4. Correlation with Clinical Data

Most of the individual phenotypic characteristics associated with virulence in other fungi studied in our* Sporothrix* strains did not present a significant correlation with the clinical data from their patients of origin (*P* > 0.05). Nevertheless, some noteworthy aspects could be observed. Regarding melanin production, we observed that the frequency of heavily melanized* Sporothrix* strains isolated from patients with spontaneous regression of sporotrichosis (93%) was significantly higher (*P* = 0.0015) than strains isolated from patients that required antifungal treatment (41%). Also, all strains that were unable to produce visible melanin pigment on colonies were isolated from cases of cutaneous sporotrichosis, meaning that strains isolated from other sites than skin produced at least lightly melanized colonies on both tested culture mediums. We also observed that eight from the fifteen strains with detectable protease activity (53%) were isolated from patients coinfected with HIV, which corresponds to 47% of HIV-infected patients who were infected with protease positive* Sporothrix* strains in contrast to 13% in the group of patients without HIV infection (*P* = 0.01). Moreover, the *P*/*z* values from strains isolated from HIV coinfected patients had a tendency to be lower than the *P*/*z* values obtained from strains obtained from HIV negative patients, although this difference was not significant (*P* = 0.07). The urease activity of strains from patients with spontaneous regression of sporotrichosis also has a tendency to be higher than the treated group but, although variable, the difference was not significant (*P* = 0.056).

### 3.5. Experimental Infection

**Figure 6:**
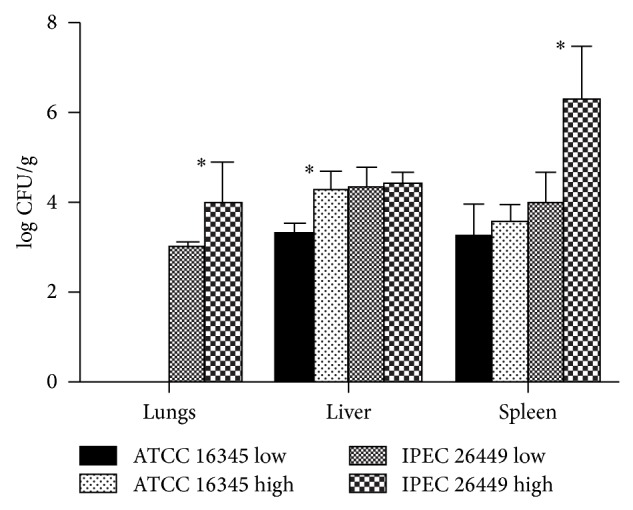
Fungal burden after an experimental infection of C57/Bl6 mice by yeast cells of one strain producing several virulence-related phenotypes (IPEC26449) and one strain with low production of these characteristics (ATCC16345). Two different inoculum sizes were used, 5 × 10^7^ (low) and 1 × 10^8^ (high)* Sporothrix* yeasts. ^*^ *P* < 0.05.

The* S. brasiliensis* IPEC 26449 strain and the* S. schenckii* ATCC 16345 were the strains with higher and lower expression of virulence-related attributes. Therefore, they were inoculated into C57BL/6 mice, using two different inoculums. No CFUs were detected in control animals injected with PBS.  Figure 6 shows the quantification of viable yeast* Sporothrix* cells in different murine organs. The inoculum size had a direct influence on the fungal burden of the infected animals, with significant differences in the spleens and lungs of the mice inoculated with* S. brasiliensis* IPEC 26449 and in the livers of mice inoculated with* S. schenckii* ATCC 16345. Comparing the infection caused by these two isolates,* S. schenckii* ATCC 16345 was not able to disseminate from the peritoneum to the lungs, whereas* S. brasiliensis* IPEC 26449 which disseminated to the three investigated organs. The fungal burden on the spleens of animals infected with the strain expressing several virulence-related phenotypic characteristics was also higher than the fungal burden caused by the strain with lower virulence-related traits, independently of the inoculum size.

## 4. Discussion

Sporotrichosis is associated with several clinical forms, with the more progressive manifestations leading to significant morbidity [2]. Some of the putative factors related to these different clinical manifestations of sporotrichosis are the fungal inoculum size, the host immune status, the depth of the traumatic inoculation of the fungus, and* Sporothrix* thermotolerance and virulence [31]. Moreover, the different species of the so-called* Sporothrix* complex have distinct virulence patterns in a murine model of infection [32] and a recent study correlated the immunogenicity and protein secretion of eight* S. schenckii*, one* S. brasiliensis*, and one* S. globosa* with their virulence in a murine model [33], but other putative virulence factors were not investigated. This same group also studied osmophilia, halophilia, pH tolerance, urease activity, casein hydrolysis, gelatinase production, DNAse, and proteinase activities on 151* Sporothrix* spp. Brazilian strains [34] with results comparable to ours. These 151 strains were further classified into the* Sporothrix* complex [35]. As in our study, there was a predominance of* S. schenckii* and* S. brasiliensis*, with a small number of* S. globosa*.

Fungal virulence is multifactorial, involving several phenotypic properties, such as host defense factors, toxins, adhesins, *α*-1,3-glucan, extracellular proteinases, estrogen-binding proteins, thermotolerance, and hydrolytic enzymes, among several other factors [17]. Furthermore, melanin can mask fungi against host immune responses, therefore contributing to fungal pathogenesis and virulence [36]. Despite their importance, the studies of these putative virulence factors in* Sporothrix* species are scarce. This is the first study that evaluates certain of these phenotypic aspects in a large number of* S. brasiliensis* strains in comparison with* S. schenckii* and* S. globosa*, and that also correlates the virulence factors with the clinical information of patients with sporotrichosis.

Our group previously showed that production of DHN-melanin and pyomelanin is highly variable among* Sporothrix* spp. strains [20, 37] and our new results strongly suggest that this variation might be related to the newly described species in the* Sporothrix* complex, since* S. brasiliensis* produced more DHN-melanin than* S. schenckii* under different experimental conditions. At present, only a few* S. brasiliensis* strains have been tested in animal models [32, 33, 38], but all of these* S. brasiliensis* strains were more virulent when compared with* S. schenckii*, which can, in part, be explained by the more rapid melanization and higher levels of pigmentation in* S. brasiliensis*. Another interesting aspect of this finding is that this species is highly associated with feline sporotrichosis [39]. Cats are highly susceptible to sporotrichosis and they can acquire the infection due to their instinct of bury urine and feces in the soil [40] where they can be infected with heavily melanized mycelial* S. brasiliensis*.

*Sporothrix* thermotolerance has long been considered an important virulence factor for several clinical manifestations of sporotrichosis [21]. For example, an analysis of* Sporothrix* spp. strains from Mexico, Guatemala, and Colombia found that isolates from Colombia, where the fixed cutaneous form of sporotrichosis predominated, had lower thermotolerance compared to strains from the other countries, where most patients had lymphocutaneous sporotrichosis [26]. Since* S. brasiliensis* has never been described outside Brazil [7, 39], it is likely that this species was not a causative agent of disease in this study. Notably, our results show that thermotolerances of* S. schenckii* and* S. brasiliensis* are similar. Moreover,* S. brasiliensis* thermotolerance does not appear to be related to clinical forms, spontaneous regression of infection, HIV-status of the patient, or sites of infection. We believe that in* S. brasiliensis* human sporotrichosis, other factors, related to the host, play an important role in the pathogenesis and outcome of infection. Our* S. globosa* strain presented very low thermotolerance, which is in agreement with the original description of this species [6]. This factor could explain the low virulence of this species in animal sporotrichosis models [32, 33]. However, we found low melanin levels in this single strain and, as there is limited published information about pigment formation in* S. globosa*, we cannot define the importance of melanization in* S. globosa* virulence.

*C. neoformans* melanin protects the fungus against high temperatures [41]. In a previous paper of our group, we have demonstrated that, although* Sporothrix* spp. can produce melanin in both mycelia and yeast forms, macroscopic evaluation of this pigment production is better achieved with cultures maintained at 22–30°C [37]. For this reason, we have chosen only to assess melanin at 25°C in the present work. We were not able to establish a connection between the degree of melanization at 25°C and thermotolerance of our strains. However, we observed a decrease in thermotolerance when selected strains were grown on presence of tricyclazole and a slight increase when cultures produced L-DOPA or L-tyrosine derived melanins, in addition to DHN-melanin. Therefore, in* Sporothrix* spp., the type of melanin that is produced by the fungus appears to influence its susceptibility to the host temperature.

The role of urease in the yeast form of* S. schenckii* has not been demonstrated. For example, a study of 49 Indian strains determined that none of the strains were able to split urea in the parasitic yeast phase; however, detectable urease activity was observed in the mycelial phase [42]. Interestingly, variable urease production was observed when the new species of the* Sporothrix* complex were described [6]. These previous studies are different from our results, suggesting that there may be a geographical pattern for urease production, an observation further confirmed by the fact that urease activity was observed in 151 Brazilian* Sporothrix* spp. strains [34]. Moreover, urease production also differs between the* Sporothrix* species, since* S. brasiliensis* produced higher urease levels than* S. schenckii*.

Urease is an important virulence factor for* C. posadasii*. This enzyme increases the pH of the exterior environment surrounding the spherules, contributing for tissue damage and exacerbation of infection [23]. Urease is also an important virulence factor in* C. neoformans* allowing the transmigration of the fungus to the brain parenchyma, a process that may be impeded by urease inhibitors [43]. The average production of urease in isolates from patients with spontaneous regression of sporotrichosis was greater than that of isolates from patients who required treatment for infection. This difference was not statistically significant, but the *P* value was very close to the significance level. Interestingly, the spontaneous regression of sporotrichosis has historically been rare [44–46]. However in Rio de Janeiro, Brazil, where* S. brasiliensis* predominates [47, 48], spontaneous regression can be observed in more than 10% of sporotrichosis cases [49]. Urease and melanin may result in an optimal activation of the immune system of patients with sporotrichosis, leading to fungal clearance without the need for antifungal treatment. Studies with a larger number of strains are needed to verify if urease production is connected to the spontaneous regression of sporotrichosis.

Protease production is also an important process in fungal infections. In* C. albicans*, protease production is associated with more severe disease [50]. We were not able to detect high protease expression in the strains of our study, which is in agreement with another study using Brazilian* Sporothrix* strains [34]. Most strains that presented a detectable protease expression were isolated from HIV-coinfected patients.

The data from the experimental infection with two different* Sporothrix* strains with different expression levels of the studied virulence factors show that, regardless of species, the higher the number of* Sporothrix* cells used in the infections, the greater the total fungal burden, confirming the correlation between the inoculum size and the pathogenesis of sporotrichosis, as previously suggested [31]. Moreover, we were able to verify an increased virulence of the* S. brasiliensis* strain that more intensely expressed several virulence factors in comparison with the* S. schenckii* strain. The higher expression of phenotypic characteristics related to virulence by* S. brasiliensis* can explain its higher virulence in murine models, when compared to* S. schenckii* [32]. However, there are factors other than expression of proteins related to virulence that should be involved in the in vivo response of* S. schenckii*, since a recent study showed that a highly virulent isolate of* S. schenckii* producing proteinase, caseinase, gelatinase, urease and DNAse was actually less virulent than a* S. brasiliensis* strain that did not produced all these factors [33]. We are aware that the number of analyzed strains is small, which can be explained by the ethical issues related to murine studies. The development of new animal experimental models such as* Galleria mellonella* [51] and* Drosophila melanogaster* [52] can be used to evaluate a larger number of* Sporothrix* strains in the future.

The well validated methods that we have leveraged in this work are similarly used at present in several other fungal models, such as* Candida parapsilosis* [53] and even* Sporothrix* spp [34]. Therefore, we believe that our approach allows for a better comparison of the results from our work with other authors. We believe that the evolving genomic information for* S. schenckii* [54] and* S. brasiliensis* [55] will allow the development of more accurate methods to assess protein expression, such as quantitative RT-PCR, which is under development by our group. Additionally, we also examined a* S. globosa* strain in this study, and there is currently no genome sequence information for this species. Therefore, this type of study with* S. globosa* and with other* Sporothrix* species without genome information still relies on the methods used in this work.

## 5. Conclusions

In conclusion, the expression of melanin, urease, and proteases in* S. brasiliensis* is higher than in* S. schenckii*, which is in accordance with other studies, including those using murine infection models. These phenotypes may be related to the clinical manifestations of sporotrichosis. Future studies, especially with a larger number of* S. globosa* strains, are necessary to gain a better understanding of these putative virulence factors role in the pathogenesis of sporotrichosis caused by different* Sporothrix* species.

## Figures and Tables

**Table 1:** Proteolytic activity of *Sporothrix* spp. strains determined by the ratio between colony diameter and azoalbumin degradation halo (*P*/*z*) after incubation at 30°C and 37°C.

Strain	Species	*P*/*z* 30°C	*P*/*z* 37°C
IPEC 23251	*S. schenckii *	1	0,81
IPEC 24372-1	*S. schenckii *	1	0,70
IPEC 25374	*S. brasiliensis *	1	0,78
IPEC 25644	*S. brasiliensis *	1	0,76
IPEC 25853	*S. brasiliensis *	1	0,59
IPEC 30650	*S. brasiliensis *	1	0,65
IPEC 31047-1	*S. brasiliensis *	0,81	0,73
IPEC 31515	*S. brasiliensis *	1	0,66
IPEC 31676	*S. brasiliensis *	1	0,74
IPEC 32004	*S. brasiliensis *	1	0,78
IPEC 33611	*S. brasiliensis *	1	0,78
IPEC 33822	*S. brasiliensis *	0,84	0,71
IPEC 33946	*S. brasiliensis *	1	0,75
IPEC 34196	*S. brasiliensis *	0,68	0,81
IPEC 34968	*S. brasiliensis *	1	0,82
